# Design of oscillatory neural networks by machine learning

**DOI:** 10.3389/fnins.2024.1307525

**Published:** 2024-03-04

**Authors:** Tamás Rudner, Wolfgang Porod, Gyorgy Csaba

**Affiliations:** ^1^Faculty of Information Technology and Bionics, Pázmány Péter Catholic University, Budapest, Hungary; ^2^Department of Electrical Engineering, University of Notre Dame (NDnano), Notre Dame, IN, United States

**Keywords:** neuromorphic computing, oscillatory neural networks, machine learning design, ring oscillators, low-power computing

## Abstract

We demonstrate the utility of machine learning algorithms for the design of oscillatory neural networks (ONNs). After constructing a circuit model of the oscillators in a machine-learning-enabled simulator and performing *Backpropagation through time* (BPTT) for determining the coupling resistances between the ring oscillators, we demonstrate the design of associative memories and multi-layered ONN classifiers. The machine-learning-designed ONNs show superior performance compared to other design methods (such as Hebbian learning), and they also enable significant simplifications in the circuit topology. We also demonstrate the design of multi-layered ONNs that show superior performance compared to single-layer ones. We argue that machine learning can be a valuable tool to unlock the true computing potential of ONNs hardware.

## 1 Introduction

The computing power of neuromorphic and artificial intelligence (AI) algorithms is greatly limited by the lack of low-power, energy-efficient hardware to run AI computing tasks. Outsourcing even the simplest AI processing primitives (such as pattern classification) to energy-efficient, specific-purpose hardware would greatly increase the prevalence and computational power of AI algorithms.

Neuromorphic analog computing elements are currently being intensely researched, as they promise significant energy savings in artificial intelligence (AI) computing tasks compared to their digital counterparts (Schuman et al., 2017). Among the many flavors of analog computing, oscillatory neural networks (ONNs) received special attention (Csaba and Porod, 2020a). This is due to the facts that (1) ONNs are realizable by very simple circuits, either by emerging devices or conventional transistor-based devices, (2) phases and frequencies enable a rich and robust (Csaba and Porod, 2020a) representation of information, and (3) biological systems seem to use oscillators to process information (Furber and Temple, 2007), likely for a reason.

Despite the significant current research efforts and the large literature, most ONNs seem to rely on some version of a Hebbian rule to define attractor states for the oscillator phases (Delacour and Todri-Sanial, 2021). The Hebbian rule is used to calculate the value of physical couplings between oscillators—such as resistances or capacitances—that define the circuit function. The reliance on the Hebbian rule turns most current ONNs into a sub-class of classical Hopfield networks, which are not very powerful by today's standards. While there are a few ONN implementations not relying on basic Hebbian rules (notably Vassilieva et al., 2011), it is likely that current ONNs do not fully exploit the potential of the hardware—due to the lack of a more powerful method to design the interconnections.

In this study, we show, using computer simulations, that a state-of-art machine learning method, namely Backpropagation Through Time (BPTT), when applied to a circuit-level model of the ONN, significantly enhances the computational power of ONNs. Our studied system is an ONN made of resistively coupled ring oscillators (Csaba et al., 2016; Moy et al., 2022), and its circuit topology is described in Section 2.1. Next, in Section 2.2 we develop the differential equations describing the circuit and show how a machine learning algorithm can be applied to design the circuit parameters. In Section 3.1, we apply the machine-learning framework for the design of an auto-associative memory and compare it to a standard Hebbian rule-based device. Section 3.1.3 furthers this concept by the design of a multi-layered network, which is a two-layer classifier and achieves superb performance compared to a single-layer device.

An AI processing pipeline typically has to process a large amount of input sensory data (such as audio, video, or text streams). These operations consume significant power, due to the sheer amount of sensory data. The ring oscillator-based ONN present here can do classification tasks in an energy-efficient way, and this way significantly increase the net power efficiency of the computing pipeline.

Overall, our study presents a design methodology that unlocks the true potential of oscillatory neural networks, overcoming the limitations imposed by simple learning rules. Additionally, the presented method allows for designing physically realizable structures: our networks rely on nearest-neighbor interactions, which is amenable to scaling, chip-scale realizations and uses significantly fewer neurons than fully connected networks.

## 2 Materials and methods

### 2.1 Resistively coupled ring oscillators for phase-based neuromorphic computation

It is well-established that the synchronization patterns of coupled oscillators may be used for computation (Csaba and Porod, 2020a). The idea of using phase for Boolean computation goes back to the early days of computer science (Wigington, 1959) and is being rediscovered these days (Roychowdhury, 2015). For neuromorphic computing, the original scheme of Izhikevich (Hoppensteadt and Izhikevich, 1999, 2000) was studied using various oscillator types and coupling schemes. A number of computing models were explored, ranging from basic convolvers (Nikonov et al., 2015) and pattern generators (Dutta et al., 2019) to hardware for handling NP-hard problems (Chai Wah Wu, 1998; Parihar et al., 2017; Moy et al., 2022).

**Figure 1:**
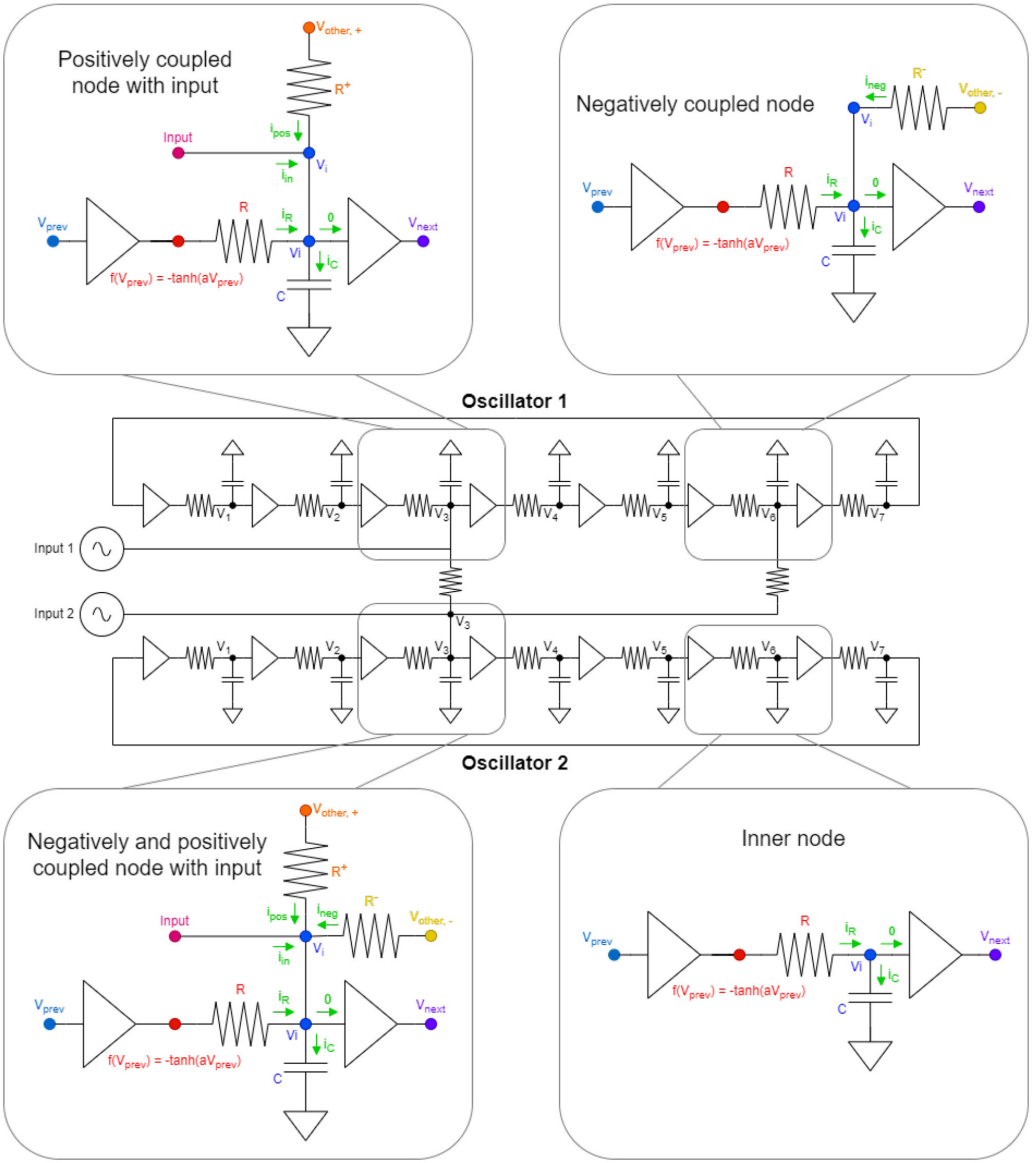
Here, in the middle, we can see a two oscillator system, coupled together in every possible way with numbered voltage nodes. The oscillators are built using seven inverters. The zoomed-in parts at the four corners are the different node types we can have in a given system. In those four schematics, the same colored nodes are at the same voltage levels and *v*_*prev*_ and *v*_*next*_ are used as the node before *v*_*i*_ and node after *v*_*i*_, respectively, because of the circular design. The green arrows symbolize the currents flowing into the given node *v*_*i*_. The 0-labeled current is a symbol for 0 current, as by definition, the input current to inverters is 0. In addition, the *v*_*other*, −_ and *v*_*other*, +_ is a symbol for the voltage node of another oscillator, which is coupled to the particular oscillator negatively and positively, respectively. The *Input*-labeled waveform generators can be anything feeding information into the system as external input currents.

Ring oscillators are among the simplest of oscillators. These devices consists only of (odd number of) inverters, capacitances, and resistances, see in Figure 1.

**Figure 2:**
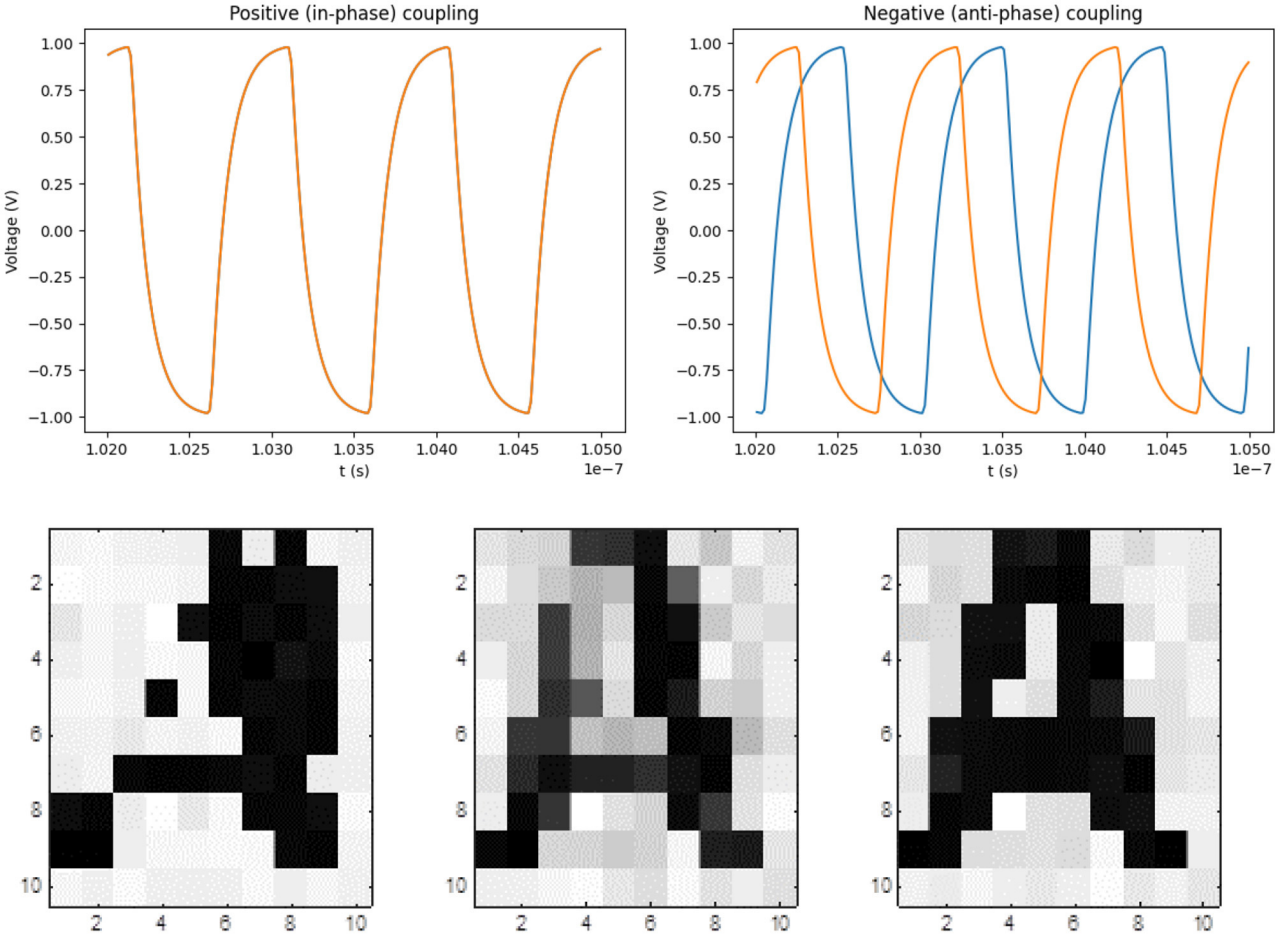
Phase-based computing by two ring oscillators: (**top left**) If *R*^−^ (or the resistance between different voltage nodes of two oscillators, say *V*_3_ and *V*_6_) dominates in the coupling, the oscillators run in phase, while (**top right**) if *R*^+^ (or the resistance between same voltage nodes of two oscillators, say *V*_3_) dominates, then anti-phase coupling is realised. The latter two are based on the criteria that coupling is realised by flowing currents and the larger the current is, the more influence the oscillators will have on one another. (**Bottom**) If phases correspond to pixels of a grayscale image, the phase dynamics may be used to converge to predefined patterns (Csaba et al., 2016). The illustrations of convergence to ‘A' are taken from Csaba and Porod (2020b).

To give a simple example of how ring oscillators compute in phase space, Figure 1 shows a two-oscillator system. Nodes that are interconnected by a resistor will synchronize in phase. If identical nodes (say *V*_3_, the 3rd voltage node of the ring oscillators) are interconnected, the oscillators will run in phase. However, in a 7-inverter ring oscillators, each node is phase-shifted by an angle of 2π/7 with respect to their neighbor. If, say, *V*_3_ of one oscillator is connected to say *V*_6_ of the oscillators, the oscillators will pull toward an anti-phase configuration. The waveforms of these two cases are illustrated in the top part of Figure 2.

A larger network of oscillators with in-phase or out-of-phase pulling resistors will converge toward an oscillatory ground state configuration, which in fact maps to the solution of the Ising problem (Moy et al., 2022). Simply put, the phase of each oscillator will converge toward a value that optimally agrees to most of the constraints imposed on the oscillator by other oscillators it is coupled to. The dynamics of the coupled oscillator network will approximate the solution of a computationally hard optimization problem. For an Ising problem, the oscillator oscillator couplings are part of the problem description, and there is no need to calculate them.

While the Ising problem is important and shows the computational power of ONNs, an Ising solver alone is not very useful for solving most real-life, neuromorphic computing tasks. A neuromorphic computing primitive (such as a classification task) does not straightforwardly map to an Ising problem. So, if the oscillator network is to be used as a neuromorphic hardware, then the oscillator weights must be designed or trained to perform certain computational functions.

Most ONNs are used as auto-associative memories, making them applicable for simple pattern recognition/classification tasks. The weights are designed based on the Hebbian learning rule (Csaba et al., 2016; Delacour and Todri-Sanial, 2021), and this is one of the cases when the Ising model easily maps to a neuromorphic computing model. In fact, the connection between Ising and Hopfield's associative models (Hopfield, 1982; Michel et al., 1989; Smith, 1999) was designed by Hopfield early on Hopfield and Tank (1985). ONNs simply use oscillator phases as the state variable of Hopfield neurons.

The Hebbian rule (and even its improved variants Righetti et al., 2006; Tolmachev and Manton, 2020) has severe limitations: the rule works best on all-to-all oscillator (neural) connections and it does not trivially support learning on a set of training examples. In addition, simple Hopfield models are not very powerful neural networks by today's standards—for example, a Hebbian-trained Hopfield network achieves mediocre results in the standard MNIST classification tasks (Belyaev and Velichko, 2020). This is why our goal in this study is to go beyond these limitations and apply state-of-the-art-learning techniques to train ONN weights. This allows us to overcome the limitations of associative (Hopfield) type models and design ONN versions of many other neural network models.

### 2.2 Machine learning framework for circuit dynamics

Our methodology is to apply Backpropagation Through Time (BPTT) (Werbos, 1990) to an in-silico model of the oscillators. We constructed a circuit model of the coupled oscillator system; the resulting ODEs are solved and the value of the loss function is calculated at the end of the procedure. By backpropagating the error, we can optimize the circuit parameters in such a way that the ONN solves the computational task defined by the loss function. Once the circuit parameters are determined via this algorithm, they can be ‘hard-wired' into a circuit (ONN hardware) for an effective hardware accelerator tool.

#### 2.2.1 Computational model of resistively coupled ring oscillators

For the sake of concreteness, we assume that our circuit consists of *n* oscillators and each oscillator is composed of seven inverters. The circuit has *k* input nodes. We construct a simple ordinary differential equation (ODE)-based circuit model based on the equations derived by Lai and Roychowdhury (2005).

Each inverter is described on a behavioral level by a *f*(*x*) = −tanh(*ax*) non-linearity connected to an *RC* delay element. This way, a seven-inverter ring oscillator is modeled by seven first-order non-linear ODEs.

The mathematical formulation consists of three parts: internal dynamics of the oscillators (due to the inverters), dynamics due to external signals (inputs), and the coupling's dynamics.

In Figure 1, there can be seen the basis of the derivation of the ODE of the circuit model. There are four types of nodes in the system and for each of them, an ordinary, first-order differential equation can be derived using Kirchoff's current law as follows:

- Most nodes are inner-nodes (bottom right part in Figure 1) in the oscillators (5 in each) and their equation is rather easy to calculate:

$$\begin{array}{l} C\frac{dv_{i}}{dt}=\frac{f\left(v_{prev}\right)-v_{i}}{R} \end{array}$$

- There can also be negatively coupled nodes (top right part on Figure 1), which are a little bit more complex than the simple inner nodes. It also has another current component flowing to *v*_*i*_, which is coming from the difference of the voltage of a different node of another oscillator and the voltage of the particular oscillator divided by the resistance between the nodes. Here, the requirement for negative coupling is that the two coupling nodes should be an odd even pair in terms of numbering of voltage nodes. Here, the equation is the following:

$$\begin{array}{l} C\frac{dv_{i}}{dt}=\frac{f\left(v_{prev}\right)-v_{i}}{R}+\frac{v_{other,-}-v_{i}}{R^{-}}. \end{array}$$

- There can be positively coupled nodes with inputs (top left part on Figure 1). Positively coupled nodes are more complex than the negative coupled nodes previously described, as it not only has an incoming current from a different oscillator but also has an external input indicated by the waveform generators on Figure 1. Note that the requirement for positive coupling between the two oscillators is to have an even even or odd odd pairing of oscillators. The particular ODE for this kind of arrangement is as follows:

$$\begin{array}{l} C\frac{dv_{i}}{dt}=\frac{f\left(v_{prev}\right)-v_{i}}{R}+\frac{v_{other,+}-v_{i}}{R^{+}}+Bu_{in}, \end{array}$$

where *B* effectively controls the amplitude of the input waveform. It is worth mentioning that input is not necessarily present for this node, so it is possible that a node only has extra current coming from positive couplings without any kind of external input.

- The most complicated node is the one having positive and negative couplings and also some input (bottom left part on Figure 1). It is basically the merger of the previous two items which is manifested in the equations as well:

$$\begin{array}{l} C\frac{dv_{i}}{dt}=\frac{f\left(v_{prev}\right)-v_{i}}{R}+\frac{v_{other,-}-v_{i}}{R^{-}}+\frac{v_{other,+}-v_{i}}{R^{+}}+Bu_{in}. \end{array}$$

Combining the previously presented knowledge, for the whole system, we can arrive at the following ODE for the collection of voltages at all the nodes, which describes all parts if we write a differential equation for every node in the system using Kirchhoff's current law and assuming only resistors as couplings:


$$\frac{dV}{dt}=\frac{1}{RC}(f(P_{\pi }V)-V)+\frac{1}{C}B^{\prime }u+\frac{1}{R_{c}C}C^{\prime }V,$$

where


$$\begin{array}{l} f\left(x\right)=-\tanh\left(ax\right), \end{array}$$

is the simplified characteristic of an inverter with some *a*∈ℝ. Furthermore, π is a permutation, such that


$$\begin{array}{l} \pi =\left(\begin{array}{ccccccc} 1 & 2 & 3 & 4 & 5 & 6 & 7\\ 7 & 1 & 2 & 3 & 4 & 5 & 6 \end{array}\right) \end{array}$$

and **P**∈ℝ^(7*n*) × (7*n*)^ is a block matrix in which for every 7*x*7 matrix block in the main diagonal there is a permutation matrix corresponding to π. This orders the voltage nodes in the ring oscillator to calculate the voltage differences arising between the two endpoints of the resistors placed in between the two inverters. **B**′∈ℝ^(7*n*) × *k*^ is the connector matrix for the inputs. The inputs are collected in *u*∈ℝ^*k*^. **C**′∈ℝ^(7*n*) × (7*n*)^ is the modified couplings matrix which is to be constructed from the real, humanly readable couplings matrix **C**∈ℝ^*n*×*n*^. The parameters *R*, *C*∈ℝ^+^ are fixed for the oscillators; meanwhile, the $R_{c}\in {\mathbb{R}}^{+}$ coupling parameters are one of the two real, to-be-learnt parameters of the system that govern the whole coupling dynamics. The other ones are the parameters gathered in **B**′, which directly relates to the amplitude of the input signal (typically a sinusoidal current generator).

The **C**_*i, j*_ is related to the couplings between oscillators *i* and *j* and the matrix is built the following way:

- All main diagonal entries are 0, as no oscillator is coupled to itself.
- All entries in the upper triangle of the matrix are corresponding to the positive (in-phase-pulling) couplings.
- All entries in the lower triangle of the matrix are corresponding to the negative (anti-phase-pulling) couplings.

The construction of **C**′ can be done easily from *C* algorithmically. As every positive coupling is between 3-3 nodes of the oscillators and every negative connection is between 3-6 nodes of oscillators, the **C**′ matrix is quite sparse. Similarly, because inputs are only fed into the 3rd node of every oscillator, the **B**′ matrix is sparse.

**Figure 3:**
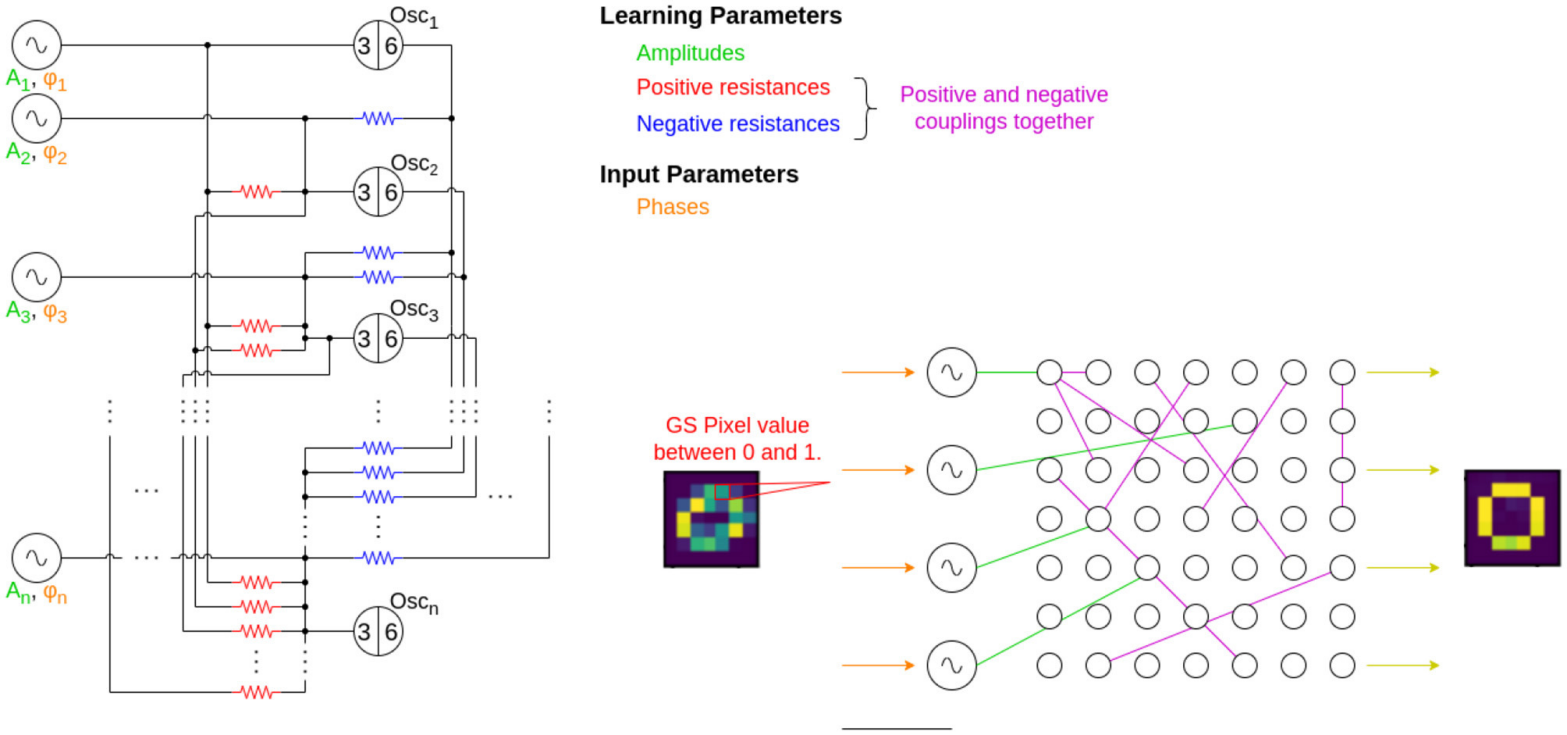
The circuit diagram of the entire computational layer. Input signal generators provide the sinusoidal signals with a phase that corresponds to an input pattern, such as pixels of an image. These generators are connected to the computing oscillators, whose phase pattern provides the solution to the problem. The 3–6 marks on the oscillators indicate the 3rd and 6th nodes in the ring oscillators' circuit. The green, red, and blue colored circuit elements' values are learned during the learning process and the phases, indicated with orange are the inputs. On the schematic figure, the purple connections indicate both the positive (red) and negative (purple) couplings. The grayscale pixel value is read from the image, converted to phase information, then the sinusoidal current generators are connected to the oscillators one-by-one. The yellow arrows show that the output is read from the oscillators and an image is formed.

The ODEs are constructed for the circuit of Figure 3, in case of a fully connected ONN. The oscillators are driven by sinusoidal current generators, and the phase of these signals carries the input. They define the initial states of the oscillators that is later changed by the couplings between the oscillators.

Each oscillator is connected by two resistors, the value of which has to be learned. The values of the coupling resistors are inversely related to the coupling parameters, which are stored in the **C** coupling matrix and the elements of this matrix are to be learned. In the equations above, *R*_*c*_ is a predefined, constant value which is the resistance scaling factor between two coupled nodes, usually around 10*kΩ*. The system learns the values in **C**. From this matrix, the **C**′ modified coupling matrix is built. The real physical coupling resistances' values between nodes *i* and *j* is given by $\frac{R_{c}}{{\text{C}}_{i,j}}$.

Similarly, the values in **B**′ are related to the input current generator's amplitude, but they are directly proportional to the real amplitude of input generators.

In the examples of the later sections, the grayscale pixel colors will typically correspond to the input phases of the current generators, and a pixel intensity from 0 to 1 is mapped to phases ϕ∈[0, π]. Similarly, the output pattern is the stable, stationary phase pattern of the oscillators.

The circuit model we use (Lai and Roychowdhury, 2005) is simpler than a SPICE-level (Simulation Program with Integrated Circuit Emphasis) circuit model, as it takes into account the transistor characteristics by a behavioral curve. The internals of the MOS transistors are neglected. This simplification is done to facilitate learning as we will explain below.

#### 2.2.2 Backpropagation for ONN circuit design

Backpropagation is the de facto standard algorithm used for the training of neural networks (LeCun et al., 2015). After each run of the neural network, the gradient of a properly defined loss function is computed with respect to the trainable parameters of the system, in an efficient manner.

BPTT (Backpropagation Through Time) is backpropagation applied to a dynamic system (i.e., an ODE-based description). The ODE is solved by a standard time-stepping technique, using discrete time. This dicretized solution may be viewed as a many-layer neural network such that one neural layer corresponds to a temporal snapshot of the system dynamics. The BPTT algorithm calculates and stores these layers (snapshots) in the forward pass of the calculation, then calculates the derivatives of an objective function with respect to trainable parameters in the backward pass.

To apply backpropagation or BPTT, a loss function (objective) function has to be defined, and this assumes a minimum value when the system is in the desired, computational state. The loss function is typically defined on the end state of the ODEs; in our case, this is the the stationary phase of oscillators at the end of the computation.

In this study, we apply BPTT to find out the circuit parameters that enable the ONN to perform useful computation. After the calculation of the gradient, a gradient descent method is used for learning, in order to minimize the loss function. Each gradient descent steps should bring the circuit parameters closer to their optimal value.

We have written our simulation code in Pytorch (Paszke et al., 2017)—the autograd feature of Pytorch makes the implementation of backpropagation and BPTT straightforward. We also used the *torchdiffeq* (Chen, 2018) package for implementing backward-differentiable ODE solvers. This external, third-party library is built upon PyTorch and provides various differentiable ODE solvers implemented for PyTorch. A particularly useful feature of *torchdiffeq* is that it can apply the adjoint method for the backward step (Chen et al., 2018) and calculate the gradients with a constant memory cost.

It must be noted that BPTT is computationally demanding for a complex dynamic system such as our ONN. The time-domain solution of a circuit model typically consists of thousands of time steps. As BPTT works by unwrapping the time-domain solution of an ODE to a many-layer neural network, the BPTT algorithm must handle a many-thousand layer network and this may yield to memory bottlenecks during the training.

Backpropagation through many layers inevitably suffers from the vanishing gradient problem (Lillicrap and Santoro, 2019). We found that our algorithm produces useful gradients up to a few thousand time steps (layers). The ONN is constructed to safely converge within this time frame.

The high computational demand of BPTT is the primary reason we have chosen a simplified circuit model for the simulation of ring oscillators. A typical Level 3 MOS model contains hundreds of parameters; while a SPICE-level simulation is straightforwardly possible even for larger circuits, learning (backpropagation) becomes computationally demanding for such models.

It is also important that BPTT supports only “*in silico*” training. The design of the ONN (i.e., the learning) takes place on a different hardware than the inference. The learning is done on a digital computer model. Once the learning is finished, the inference is done on a dedicated hardware that uses the computer-learned circuit parameters. Online learning is not possible this way, but our goal is to realize efficient, “hard wired” hardware for inference.

**Figure 4:**
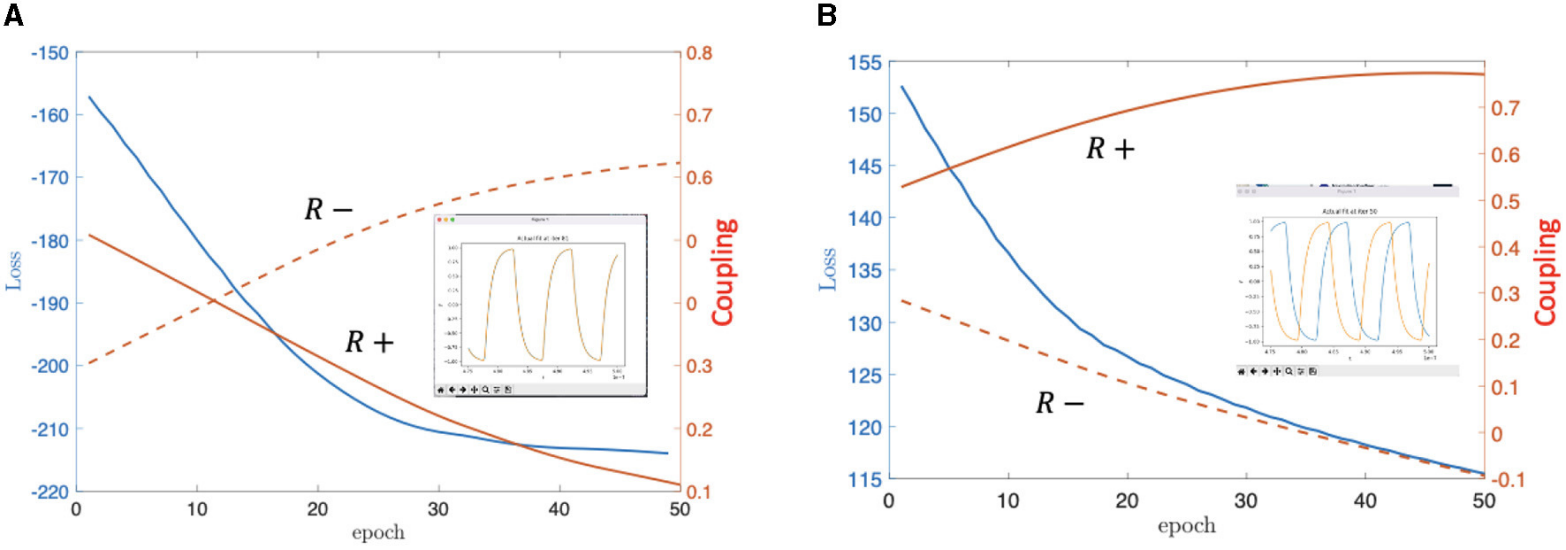
On **(A)** we can see the simulation's result for the positively coupled oscillators, meanwhile on **(B)**, there is the same for the negatively coupled 2-oscillator system. The loss changed in both cases from high value to low value. In addition, the orange curves are indicating the learning parameters' values contained in **C** and not the real values of the resistors. Note that in **(B)**, the parameter value corresponding to “R-” is going below 0, which would mean a negative resistance because of the connection of the parameters in **C** to the physical parameters, but this is only the mathematical solution, for a given simulation, the parameters were clamped to be non-negative and if they hit zero, the connection removed. **(A)** In-phase coupling learned. **(B)** Anti-phase coupling learned.

Figure 4 exemplifies the learning procedure for the two-oscillator system of Figure 2. We selected the loss function of the system as the dot product of the oscillator waveforms, which is a standard choice for this type of problems. The loss should be maximized (minimized) for in-phase (anti-phase) coupling. Such mean-square machine learning algorithm adjusts the value of the *C*_*i, j*_ parameters (and the coupling resistors) until this desired phase configuration is reached.

This method can be straightforwardly generalized to achieve convergence toward more complex patterns. If the loss function aims to maximize the dot product of waveforms between same-colored pixels and minimize them between different-colored ones, then the phase pattern can converge toward any prescribed image. If the phase pattern made to converge toward different patterns for different inputs, then the ONN will act as an associative memory.

Since the Machine Learning (ML) technique is designing a physical circuit, safeguards were taken not to arrive to unrealizable circuit parameters such as negative resistances or exceedingly strong couplings that would quench oscillations. This was done by clipping the values after each learning step to a given interval.

## 3 Results

### 3.1 ONN-based pattern association on the MNIST dataset

We have chosen the standard MNIST database for testing the associative capabilities of our system. Since the BPTT algorithm is computationally demanding, we made a few simplifications. We downsampled the initially 28 × 28 pixel-sized picture from MNIST to have either 14 × 14 or 7 × 7 size using average pooling. This allowed us to have a reduced dimension for the input images, and also keep the necessary information because of the average pooling. In addition, 14x14 MNIST images are still recognizable as a human, so it allowed us to easily recognize if some patterns are easier for the algorithm to distinguish from the others.

#### 3.1.1 Baseline: ONN-based associative memory with Hebbian learning

The simplest, well-studied ONN-based associative memory can be designed by the Hebbian rule. If we want the phase pattern to converge toward ξ or η for inputs resembling to ξ or η, then the weights that realize this associative memory are:


$$C_{ij}^{cpl}=\frac{1}{2}(\xi_{i}\xi_{j}+\eta_{i}\eta_{j}),$$

where ξ_*i*_ and ξ_*j*_ is the *i*-th and *j*-th element of the pattern ξ, and η_*i*_ and η_*j*_ is the *i*-th and *j*-th element of the pattern η, respectively.

The rule assumes all-to-all couplings, making a larger-scale network hard to physically realize.

In our Hebbian learning scheme, the weights were determined initially in a single-shot formula, and in our test case, we applied the learning to optimize the value of base coupling resistances, *R*_*c*_, and the parameters in **B**′, which are the amplitudes of input current generators.

The inner *RC* time constant of the ring oscillators was 2.0·10^−10^ s, which translates into a 500 MHz oscillation frequency (time period *T* = 2 ns). The total simulation time for the network is 500 ns. The phase pattern is calculated from the last 300 ns window, so convergence is achieved after less than 100 oscillation cycles or 200 ns.

#### 3.1.2 ONN-based associative memories with all-to-all and nearest-neighbor coupling

The same functionality that is realized by Hebbian learning can be achieved by the BPTT method. The loss function we selected was:


$$\begin{array}{l} L=\frac{1}{n}\overset{n}{\underset{k=0}{\sum }}{\left(O_{k}-T_{k}\right)}^{2}, \end{array}$$

where *O*_*k*_ is the pattern calculated from the output of the oscillators for the *k*-th input in the batch and *T*_*k*_ is the ground truth for the same, which were ideal patterns of “0” and “1”. In the above formula, *n* is the size of the batch used for learning.

**Figure 5:**
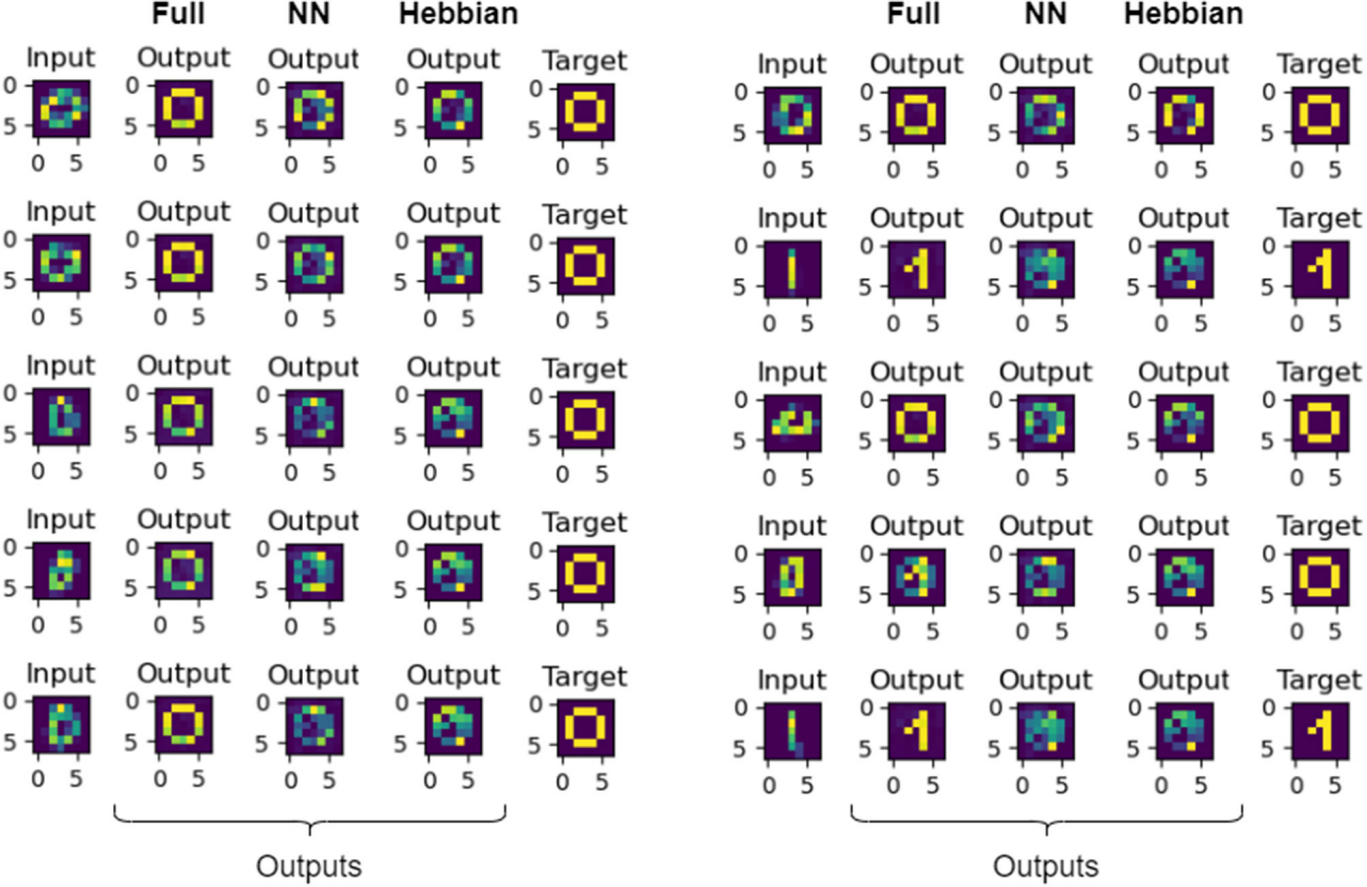
Here, we can see the comparison of the results of the fully connected, nearest neighbor connected, and the Hebbian-learned based networks. The two blocks of five inputs are shown side-by-side. The first column in each block corresponds to the input digit, the next three is the output of the systems (in order from left to right: fully coupled, nearest neighbor coupled and Hebbian-based). The last column in both blocks shows the target digit. It is apparent that the fully connected system worked best, but even our proposed, nearest neighbor connected topology was outperforming the Hebbian-based architecture.

Figure 5 compares results from the Hebbian- and BPTT-based designs. It is visually apparent that the BPTT-based design associates to the right pattern from very much distorted patterns. For the experiments seen in Figure 5, we downscaled the images from 28 × 28 to 7 × 7 which distorted many of the inputs. It helped speed up the computations, because an all-to-all coupled 728 oscillator system would result in almost 620000 resistors. This is hard to physically realize.

Most importantly, the BPTT-based design allows the design of sparsely interconnected circuit topologies. We used it to design the *C*_*ij*_ matrix of associative memory assuming only nearest neighbor interconnections. The nearest-neighbor interconnected, BPTT-designed network outperforms the fully interconnected Hebbian network, even if the number of trainable parameters in the system (≈8*n* vs. $\frac{1}{2}n^{2}$) is significantly less. The qualitative results of this comparison can be seen in Figure 5.

The result that a nearest-neighbor (NN) interconnected (BPTT-designed) network outperforms the (Hebbian-designed) fully connected network is important. In a fully connected ONN, the number of connections grows quadratically with the number of oscillators, making large, fully connected circuits unrealizable. Only locally connected architectures yield to scalable, physically realizable ONN circuits.

Quantitatively, the results of the different approaches for the whole dataset *S* = {0, 1} can be seen in Table 1.

**Table 1:** The MSEs of all the elements from the set and their respective ground truths for the different methods in case of the associative learning.

**Method**	**Hebbian**	**Proposed fully connected**	**Proposed NN connected**
#Params	1,176	2,352	312
MSE	0.068	0.020	0.047

It is apparent that the fully connected network performed the best but even the nearest neighbor connected layer is good enough to beat the Hebbian learning in terms of quantitative association.

### 3.2 Multi-layered ONNs for classification on the MNIST database

**Figure 6:**
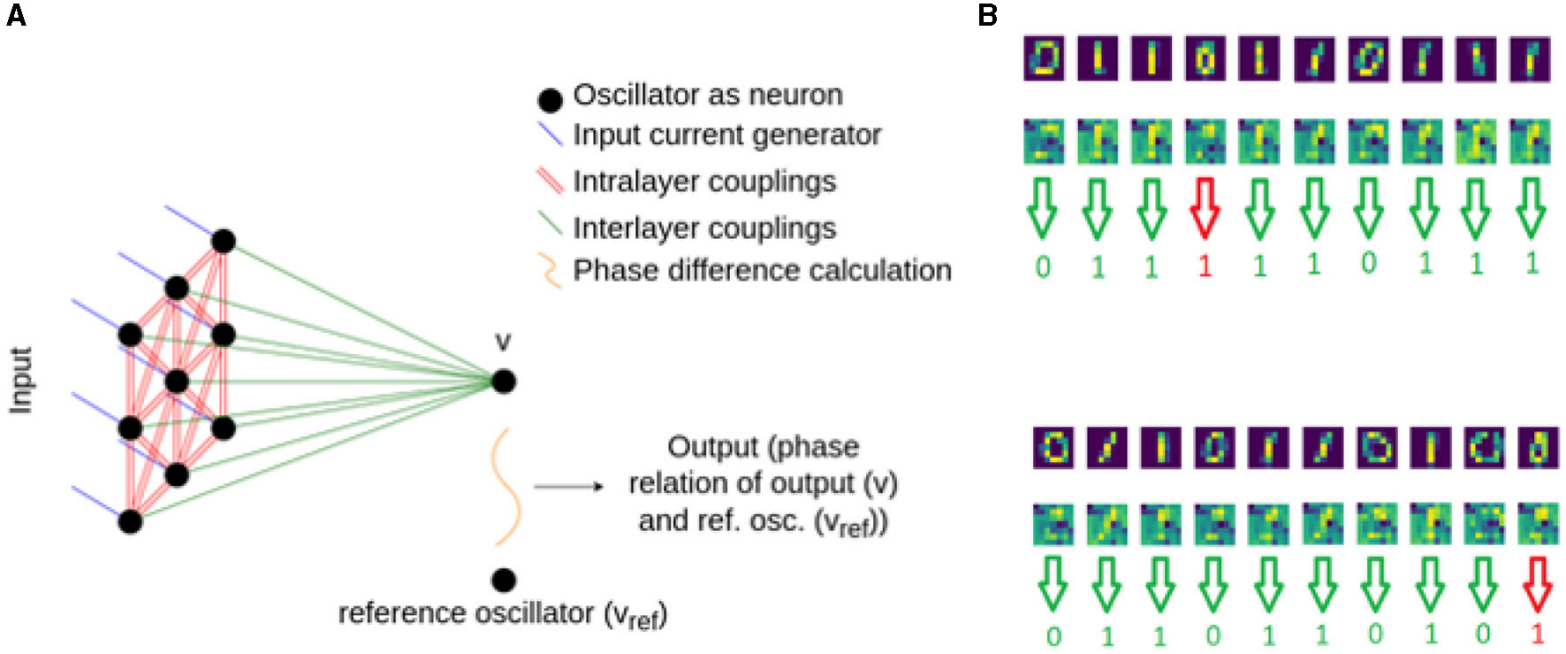
A simple two-layer classifier showing also the patterns forming in the hidden layer.

Single layer associative memories are not particularly efficient for classifying all the 10 MNIST classes, as there are strong correlations between the different digits. The BPTT method does not require the oscillators of the network to converge to a prescribed phase pattern so there is no need to use associative memory for classification. For this reason, we investigate a simple multilayer ONN, where the second layer is a single oscillator connected to all oscillators of the input layer as illustrated in Figure 6.

**Figure 7:**
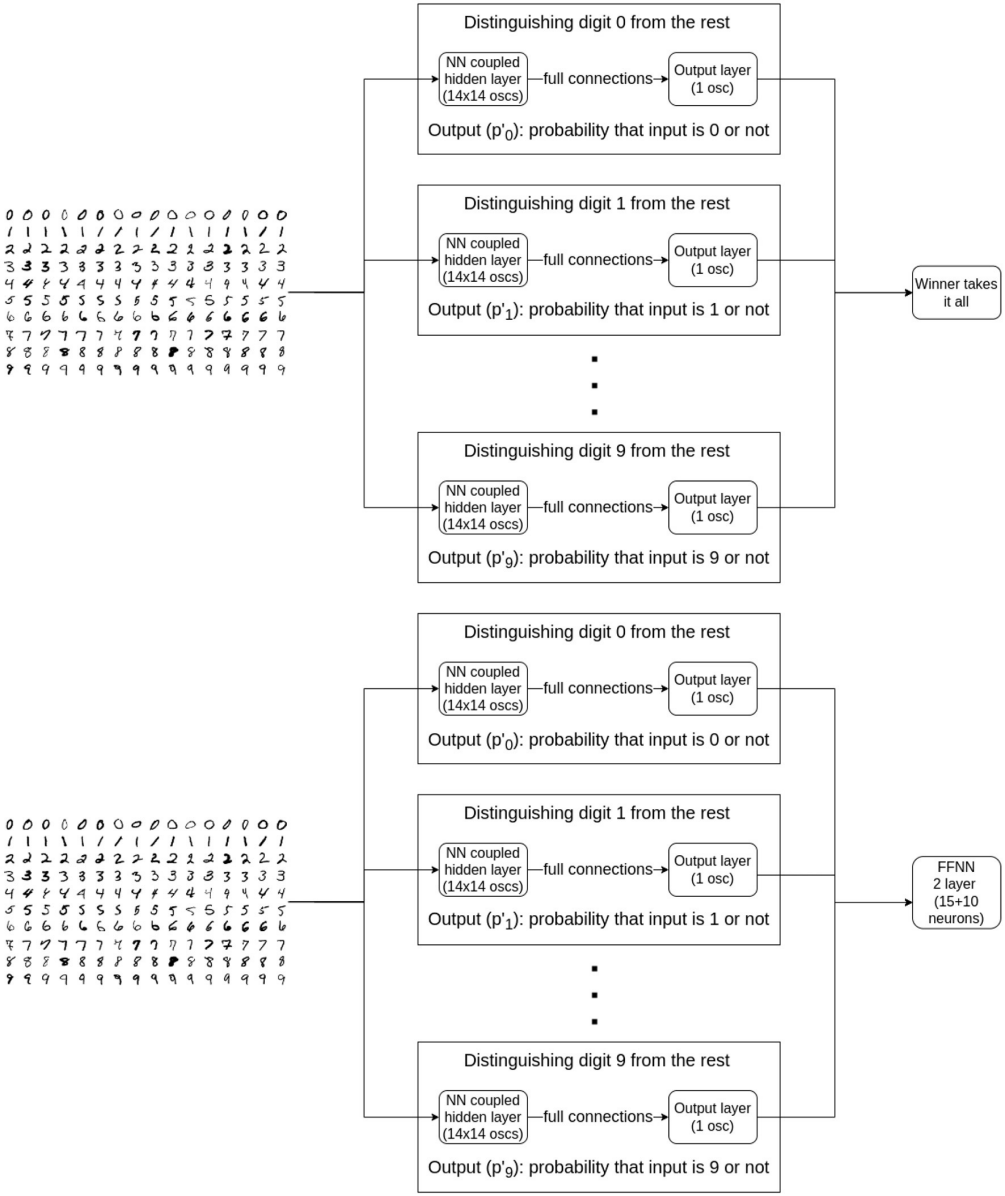
Two of the three tested architectures for the time-independent MNIST classification are shown. Both consist the individually trained, nearest-neighbor-connected subnetworks which were designed to distinguish between a single class and the rest of the classes using binary cross-entropy loss function. The top block diagram describes the algorithm where to pick the prediction, we took the maximum of individual network output probabilities. The more sophisticated version can be seen on the bottom block diagram. Here, we took the output probabilities of the individual classifiers and fed them as inputs to a small, regular FFNN and trained it as if it were a 10-class classification problem using cross-entropy.

We used architecture in Figure 6 in various ways: for binary classification, one hidden layer and a single output yielded decent results, as described in Section 3.1.3.1. For classifying all 10 digits, we trained 10 blocks (Figure 6), each responsible for recognizing one particular digit, and evaluated them with a winner takes all decision (see Section 3.1.3.2). Finally, we swapped the “winner takes it all” method for a small, MLP (multi-layered perceptron) model, composed of just a few neurons. This architecture is shown in Figure 7 and discussed in Section 3.1.3.3.

#### 3.2.1 Binary classifiers with a single output

The two-layer classifier is shown in Figure 6. The phase of the output oscillator carries the classification result: we compare the output oscillator's phase with a reference oscillator's phase and maximize (minimize) their phase difference for one (or the other) pattern.

Since the optimal oscillator couplings are discovered by the BPTT algorithm, this device does not necessarily work as an associative memory. The phase patterns appearing in the hidden layer are non-intuitive, albeit occasionally they vaguely resemble the images to be recognized.

That having been said, without any apparent, clearly visible structure in the hidden layer, the network was predicting the two classes at a 98% success rate. The predictions made on some images are present in Figure 6.

#### 3.2.2 10-digit classifier using a winner takes it all output

Classifying all 10 digits is a significantly more difficult task than the basic binary classifier and requires many more oscillators. Training a large number of oscillators simultaneously is prohibitively difficult with our method. Instead, training everything at once, we trained 10 separate blocks (subnetworks), each being responsible for recognizing one particular digit - as seen on Figure 7. The blocks themselves are nearest-neighbor connected. The individually trained networks are connected to a winner-takes-all circuit that decides the result of the 10-class classification.

**Figure 8:**
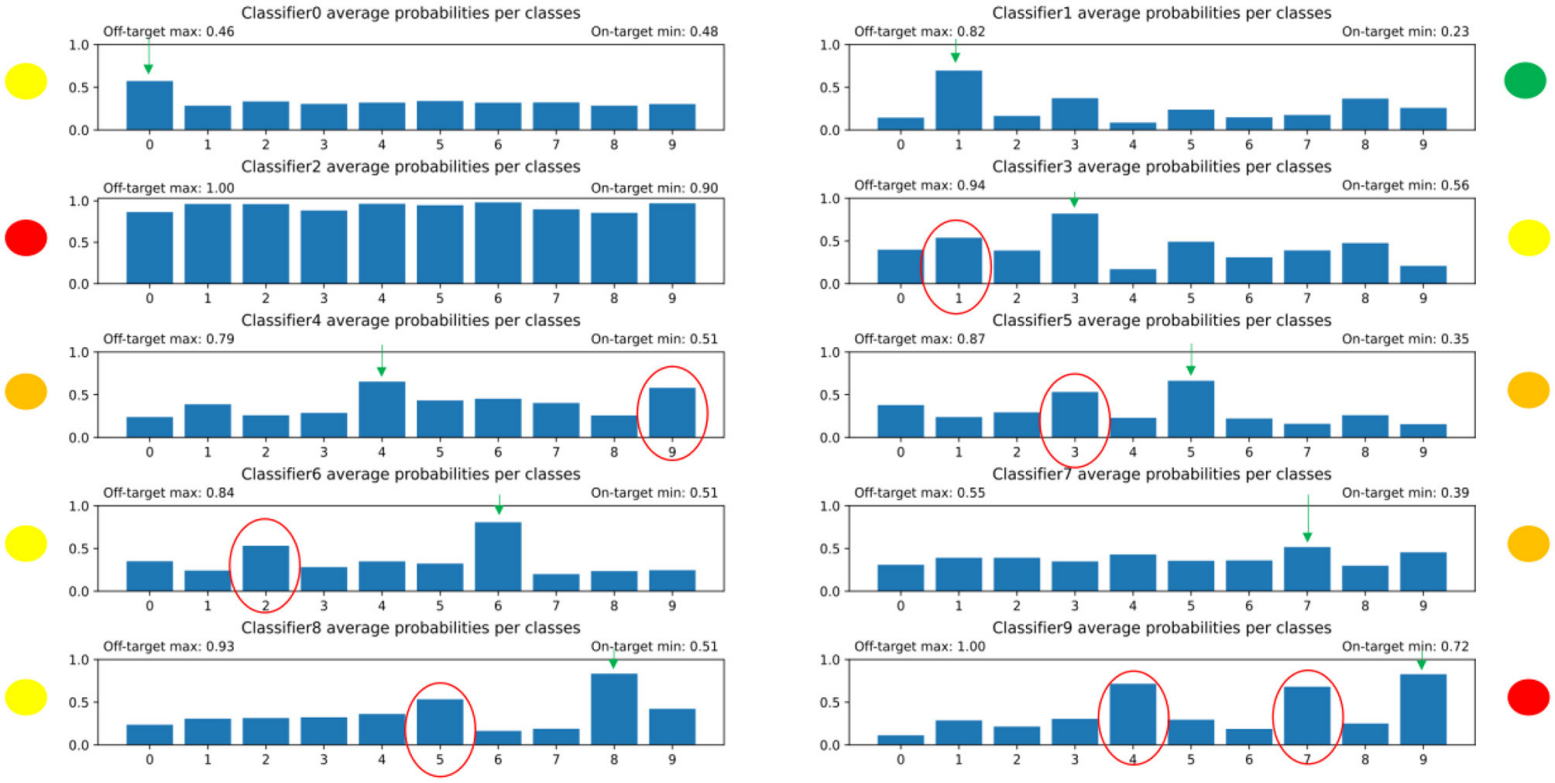
The distribution of predicted average probabilities for the individual, competitive networks in the winner takes it all model. The red-circled bars are those that on average were too high as probabilities because the given subnetwork should not have high values for that specific digit. The green arrows indicate which bar should be the highest. The yellow, orange, and red dots near the plots indicate how well the subnetwork managed to solve its task. It can be seen that this had to be improved.

**Figure 9:**
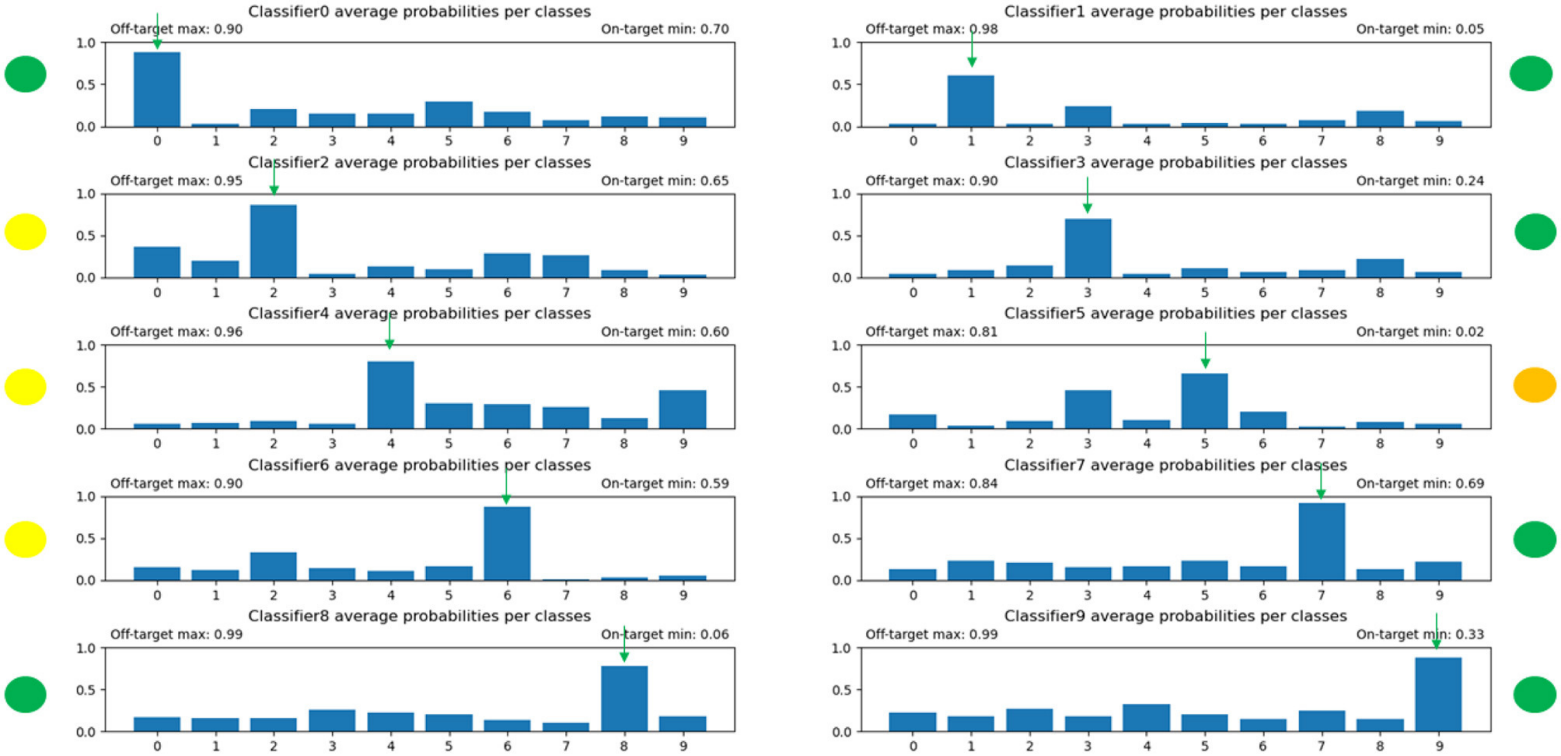
The distribution of the predicted average probabilities after extensive training. It is evident that the distributions improved, but there are still some outliers where the non-target digits are having too high probabilities.

The results of the distribution of average values of the predictions of each individual, competitive network can be seen in Figure 8. After further training, the output probabilities of the individual networks were improving, but still not aligned perfectly to the desired distributions as can be seen on Figure 9. Some digits predicts a high likelihood for the wrong classes. Using the winner takes it all algorithm (i.e., the decision is made by the ONNs using the 10 output likelihoods from the architectures and the highest one is the winner), we achieved an accuracy around 70%. To put this number in context, random guessing would be 10 %, but the state of art for MNIST digits is above 99%.

#### 3.2.3 10-digit classifier using a trained second layer

**Figure 10:**
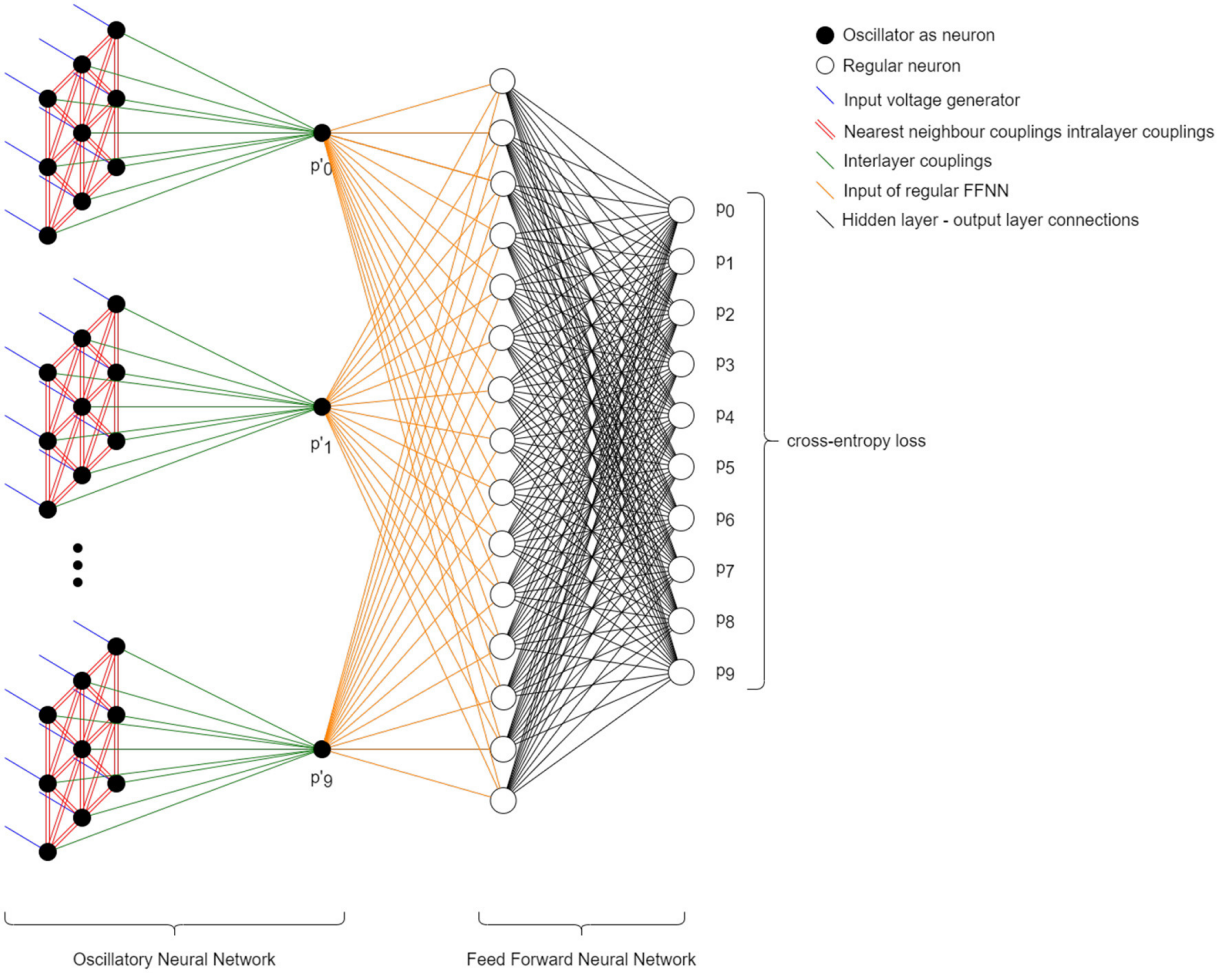
A network architecture with ONN layers as preprocessors and a traditional neural network postprocessing the results. The easy-to-train output layer significantly improves classification accuracy.

Instead of the winner takes it all decision, we used a simple multilayered perceptron at the end to improve classification accuracy. It consists of 2 layers: one hidden layer and one output layer. The hidden layer has 15 and the output has 10 neurons. The structure of this new setup can be seen on Figure 10. This means that only 325 extra parameters are introduced, which is negligbly small compared to the roughly 16000 parameters of the ONN layers.

The reason we have chosen a traditional Feed Forward Neural Network (FFNN) layer to improve accuracy are entirely practical: such FFNN is easy to train and we could train it straightforwardly after all the ONN blocks were designed. We emphasize that this conventional NN layer does not alter our conclusions, and the vast majority of the computation is still done by the ONN network. It is worth to note that there are very few multi-layered ONNs in the literature [a few examples are Karg et al. (2021), Abernot and Aida (2023), or Velichko et al. (2019)].

Using the outputs of the competitive networks as inputs to this small neural network, we managed to reach 96.7% predictive accuracy. This is excellent accuracy for a network of this size. We implemented feedforward (perceptron) neural networks with identical number of parameters, and such networks typically reach 93–95% accuracy. While the MNIST problem is solved with fairly trivial networks with accuracy approaching 100%, these networks are using hundreds of thousands of parameters and we only had 20000 parameters in our training scheme.

We emphasize that in terms of computation workload, the heavy lifting in this architecture is done by the ONN-based preprocessing layer—the output layer contains a small number of parameters and it is a very small-scale neural network by any standard. The output layer is there since it is easily trainable so it can maximize network performance at low training cost. The power consumption of the network is dominated by the ONN, and so the entire architecture benefits from the energy-efficient ONN operation. This result hints that ONNs excel as first layers (preprocessing layers) in an AI pipeline.

Integrating oscillatory neural networks (ONNs) with compact traditional neural networks, resembling perceptrons, presents a promising avenue to leverage their combined strengths. ONNs can perform complex, dynamic computation but they are difficult to train. Perceptrons (what we used here) can be easily trained to specific tasks, but they have limited computational might. Putting ONNs close to sensory inputs, where most input data has to be handled (and where most power is consumed), and refining the computing function, a higher level with an easily trainable layer could harness the best of both worlds and yield the best overall power efficiency for the network.

### 3.3 Comparison of ONN classifier architectures

The Table 2 quantitatively summarizes some key findings of our study. Most importantly, the ONN-based network outperforms a standard FFNN with the same amount of parameters. This is not entirely surprising for two reasons: one is that ONNs are recurrent neural networks, exhibiting complex dynamics, unlike an FFNN. The other reason is that ONNs carry information in the phase, frequency, and amplitude of their signals, while a standard neuron outputs only one value (which is usually a static voltage in a hardware realization). So one may expect that an ONN, if properly trained, may be able to perform more complex functions with same number of neurons (processing units).

**Table 2:** The quantitative comparisons of binary and multi-class classifiers with the parameter count indicated.

	**Binary classifiers**		**Multiclass classifiers with oscillators**			**Benchmark**
**Method**	**Fully**	**NN**	**FFNN-like**	**Winner takes all**	**Augmented**	**Perceptron FFNN**
#Param	38,416	1,600	40,180	16,000	16,325	16,363
Perf. (%)	98	98	72.3 (70–75)	66.7 (65–70)	95.1 (93–97)	94.4 (93–95)

The “Augmented” network is the MLP-augmented network, using the two-layered MLP as the last function instead of the “Winner takes all”. For the binary classifiers, the 98% performance is the worst case scenario. The multiclass classifiers and the benchmark model have ranges between the worst and best performances. The differences are the random initial values for the learning parameters.

As a back-of-envelope calculation, if we assume a hardware similar to Moy et al. (2022), a single ring oscillator in our circuit would consume about a picojoule of power per inference, so the net power consumption of the competitive multi-layered device (with 20,000 ring oscillators) is estimated to be 4 × 10^−8^ joules/inference. Highly optimized lightweight hardware neural networks achieve in the ballpark of 1μ J/inference for a similar problem (Dressen, 2023). GPU-based networks are usually designed to achieve higher accuracy at much higher power consumption, even if state-of-art GPU chips are manufactured using a much more advanced technology node than the work of Moy et al. (2022). Overall, these numbers suggest that building the ONN we studied here by simulations would give orders-of-magnitude improvements in power efficiency compared to state-of-art solutions.

In conclusion, the ONN is not only more economical in terms of parameters but does its job with a significantly higher power efficiency than the equivalent digital or software implementation.

## 4 Discussion

In this study, we introduced an in-silico method to design ONNs. We build a computational model of the ONN, apply BPTT techniques on this model and determine circuit parameters automatically using the BPTT training algorithm.

In the current literature of ONNs, Hebbian learning rules are used almost exclusively to realize associative memories or classifiers. The reader is referred to Núñez et al. (2021), Abernot and Aida (2023), Delacour and Todri-Sanial (2021), and Nikonov et al. (2015) and to the references therein. The performance and the capabilities of a simple Hebbian rule is quite limited when compared to modern ML algorithms. One may suspect that if an ONN is designed by Hebbian rules, the capabilities of the ONN will be more likely constrained by the learning rule, and not by the ONN hardware itself.

The BPTT-based design allowed us to use simulations for exploring the limits of ONN hardware without the limitations imposed by the simplicity of the training algorithm. We indeed found that the state-of-art learning method significantly increased the accuracy of the ONN classification, and this is one main result of this study.

Another key benefit of our method is that it allows the design of ONNs that is amenable to circuit realization. For example, we have shown that a nearest-neighbor-connected ONN that is designed by BPTT can outperform a fully connected Hebbian-trained device. Since only locally connected ONNs are scalable to meaningful problem sizes, this discovery opens the door to physically realizable ONNs, which perform complex processing functions without an unfeasibly high number of interconnections. In addition to that, the BPTT method may also be used to design higher-interconnected networks (such as all-to-all connected ones) that greatly outperform their Hebbian counterparts.

Another result of the study was the design of multi-layered ONN devices, of which very few exist in literature. The ONN first layer (preprocessing layer) is followed by a simple perceptron-based layer, and classification accuracy of 95 % is reached. In line with expectations, we find that multiple layers significantly enhance the capabilities of the network. We also find that the number of circuit parameters we had to train is smaller than the number of parameters of a similarly performing standard FFNN. This means that the ONN is more economical in terms of parameters. This benefit appears on top of the benefit in power efficiency: the analog ONN circuit dynamics does its job from the fraction of the power of a number-crunching digital solution.

Our design method is not without hindrances. One of its drawback is that it is not applicable to online training, the ONN must be trained on its computer model (*in silico*) and then the weights are hard-wired into a hardware circuitry. This is acceptable for an edge-AI accelerator, where energy-efficient operation is the main figure of merit. Further research is required to find training methods that would allow continuous, online learning.

## Data availability statement

The raw data supporting the conclusions of this article will be made available by the authors, without undue reservation.

## Author contributions

TR: Conceptualization, Investigation, Methodology, Software, Visualization, Writing – original draft, Writing – review & editing. WP: Conceptualization, Funding acquisition, Supervision, Writing – review & editing. GC: Conceptualization, Funding acquisition, Methodology, Supervision, Writing – original draft, Writing – review & editing.
